# Minimized Extracorporeal Circulation Is Associated with Reduced Plasma Levels of Free-Circulating Mitochondrial DNA Compared to Conventional Cardiopulmonary Bypass: A Secondary Analysis of an Exploratory, Prospective, Interventional Study

**DOI:** 10.3390/jcm11112994

**Published:** 2022-05-25

**Authors:** Thomas Zajonz, Christian Koch, Jan Schwiddessen, Melanie Markmann, Matthias Hecker, Fabian Edinger, Götz Schmidt, Andreas Boening, Michael Sander, Emmanuel Schneck

**Affiliations:** 1 Department of Anesthesiology, Operative Intensive Care Medicine and Pain Therapy, University Hospital of Giessen and Marburg, 35392 Giessen, Germany; thomaszajonz@hotmail.de (T.Z.); jan.schwiddessen@chiru.med.uni-giessen.de (J.S.); melanie.markmann@chiru.med.uni-giessen.de (M.M.); fabian.edinger@chiru.med.uni-giessen.de (F.E.); goetz.f.schmidt@chiru.med.uni-giessen.de (G.S.); michael.sander@chiru.med.uni-giessen.de (M.S.); emmanuel.schneck@chiru.med.uni-giessen.de (E.S.); 2 German Center for Infection Research (DZIF), Partner Site Giessen-Marburg-Langen, 35392 Giessen, Germany; 3 Department of Respiratory and Critical Care Medicine, University of Giessen and Marburg Lung Center (UGMLC), University Hospital Giessen, Justus Liebig University of Giessen, Germany Member of the German Center for Lung Research (DZL), 35392 Giessen, Germany; matthias.hecker@innere.med.uni-giessen.de; 4 Department of Cardiac and Vascular Surgery, Justus Liebig University Giessen and University Hospital Giessen and Marburg, 35392 Giessen, Germany; andreas.boening@chiru.med.uni-giessen.de

**Keywords:** cardiac surgery, DAMPs, SIRS, inflammation, coronary artery bypass graft

## Abstract

The use of minimized extracorporeal circulation (MiECC) during cardiac surgery is associated with a reduced inflammatory reaction compared to conventional cardiopulmonary bypass (cCPB). Since it is unknown if MiECC also reduces the amount of free-circulating mitochondrial DNA (mtDNA), this study aims to compare MiECC-induced mtDNA release to that of cCPB as well as to identify potential relations between the plasma levels of mtDNA and an adverse outcome. Overall, 45 patients undergoing cardiac surgery with either cCPB or MiECC were included in the study. MtDNA encoding for NADH dehydrogenase 1 was quantified with quantitative polymerase chain reaction. The plasma amount of mtDNA was significantly lower in patients undergoing cardiac surgery with MiECC compared to cCPB (MiECC: 161.8 (65.5–501.9); cCPB 190.8 (82–705.7); *p* < 0.001). Plasma levels of mtDNA showed comparable kinetics independently of the study group and peaked during CPB (MiECC preoperative: 68.2 (26.5–104.9); MiECC 60 min after start of CPB: 536.5 (215.7–919.6); cCPB preoperative: 152.5 (80.9–207.6); cCPB 60 min after start of CPB: 1818.0 (844.2–3932.2); all *p* < 0.001). Patients offering an mtDNA blood concentration of >650 copies/µL after the commencement of CPB had a 5-fold higher risk for postoperative atrial fibrillation independently of the type of cardiopulmonary bypass. An amount of mtDNA being higher than 650 copies/µL showed moderate predictive power (AUROC 0.71 (0.53–071)) for the identification of postoperative atrial fibrillation. In conclusion, plasma levels of mtDNA were lower in patients undergoing cardiac surgery with MiECC compared to cCPB. The amount of mtDNA at the beginning of the CPB was associated with postoperative atrial fibrillation independent of the type of cardiopulmonary bypass.

## 1. Introduction

Over the past decades, cardiac surgery has undergone a continuous evolution regarding the optimization of surgical, anesthesiologic, and perfusion techniques. Still, the majority of cardiac surgery has to be performed with the use of a cardiopulmonary bypass (CPB) in order to guarantee sufficient perfusion, oxygenation, and decarboxylation of the body. Several technical upgrades have been introduced to improve the safety and efficiency of conventional CPB (cCPB) systems [1,2]. Nevertheless, the introduction of the minimized extracorporeal circulation (MiECC) has gained widespread interest due to its protective effect on myocardial, renal, and neurological functions [3,4,5]. MiECC is mainly defined as a closed circulatory system with a reduced priming volume containing biologically inert blood contact surfaces [1]. As a result of the minimization of the cardiopulmonary circuit, recirculation and blood–air contact are efficiently minimized, leading to a decrease in blood lysis and inflammation [6]. For example, the MiECC-induced extent of interleukin-6 (IL-6) release can be compared to off-pump cardiac surgery [7,8,9].

However, even though it is a reasonable target of interest, it remains unknown if the release of free-circulating mitochondrial DNA (mtDNA) differs after MiECC, respective to cCPB. It is well-described that mtDNA acts as a damage-associated molecular pattern (DAMP) and is therefore closely connected to an activation of the inflammatory and coagulatory response [10,11]. During cardiac surgery, mtDNA activates toll-like receptor 9, which in turn plays a pivotal role in neutrophil and platelet activation [12,13,14]. It is released by various cells such as the endothelium and neutrophils, and is therefore part of the innate immune response. Further, mtDNA contributes to the formation of neutrophil extracellular traps [10,11]. Caused by its broad physiologic function during inflammatory conditions, increased levels of mtDNA have already been connected to sepsis, trauma, and malignant diseases [10,15,16]. Moreover, cardiac surgical patients suffering from a systemic inflammatory reaction showed an elevation of mtDNA in their peripheral blood, which might be associated with a higher incidence of postoperative atrial fibrillation (POAF) [13,17,18].

Since MiECC reduces the severity of the CPB-induced inflammatory reaction, it can be assumed that it is also associated with a decrease in mtDNA release. For this reason, we hypothesized that the amount of free-circulating mtDNA is higher in patients undergoing cCBP compared to MiECC. Secondary aims of this study are the identification of potential associations between the amount of mtDNA and the occurrence of common adverse events after cardiac surgery (infections, readmission to the intensive care unit (ICU), acute kidney injury (AKI), and POAF).

## 2. Experimental Section

### 2.1. Study Design

Due to a lack of pre-existing data on mtDNA release during MiECC, this study was designed as an explorative study and was therefore performed as a secondary analysis, based on a single-center, prospective, interventional study. The primary study included 90 patients, of which 45 patients were hemodynamically optimized with a goal-directed treatment protocol. To exclude a therapeutic effect of the hemodynamic intervention, the present study analysis included only the non-interventional patients of the primary study (*n* = 45).

All patients received coronary artery bypass graft (CABG) surgery at the University Hospital of Giessen. The local ethics committee approved the primary study as well as the secondary data analysis (Justus-Liebig-University of Giessen; trial code: 30/16; approval of amendment on 18 July 2018). The primary study was registered in the German Clinical Trials Register (trial code: DRKS00010959). Both the original study and its secondary analysis were performed in accordance with the Helsinki Declaration, and all methods and results are presented in accordance with the Strengthening the Reporting of Observational Studies in Epidemiology (STROBE) guidelines.

Between September 2016 and December 2018, all consecutive patients of legal age who were scheduled for elective CABG surgery were enrolled in the study. All patients signed informed consent forms. Exclusion criteria included severe arrhythmia, severe heart or renal failure (left ventricular function ≤30%, Kidney Disease: Improving Global Outcomes (KDIGO) stadium >2, respectively [19]), current pregnancy or nursing, simultaneous participation in other interventional studies, history of autoimmune or infectious diseases, and the need for immunomodulatory medication.

The primary study goal objective was to quantify the amount of free-circulating mtDNA quantified by quantitative polymerase chain reaction (qPCR). Secondary outcome parameters included ICU readmission, postoperative infections (defined as pneumoniae, catheter-related bloodstream infections, and urogenital infections), POAF, and AKI (defined as ≥stage II by the definition of KDIGO [19]).

### 2.2. Management of Cardiopulmonary Bypass

The non-interventional study patients received standard anesthetic induction with sufentanil (0.25–0.5 µg/kg), etomidate (0.1–0.2 mg/kg), and pancuronium (0.05–0.1 mg/kg), while narcosis was maintained with propofol (3 mg/kg/h) and sufentanil (0.3–1 µg/kg/h). Central venous access was implemented into the internal jugular vein, while the radial artery was used for blood pressure monitoring.

Both cCBP and MiECC were based on the S5 Heart-Lung Machine (LivaNova, London, UK) with a membrane oxygenator (CAPIOX^®^ FX 15 Oxygenator (Terumo, Tokyo, Japan) for MiECC and an Inspire^®^ 6 F Oxygenator (LivaNova, London, UK) for cCPB, respectively), and an in-house specific perfusion tubing system (LivaNova, London, UK). For cCPB, a roller pump and an additional reservoir (both LivaNova, London, UK) were used, while MiECC was run with a centrifugal pump and without a reservoir or hemofilter. Priming solution was produced with 1 L of crystalloid (Sterofundin Iso^®^, Braun, Melsungen, Germany), 250 mL of mannitol 15% (Seraq-Wiessner, Naila, Germany), and 50 mL of albumin 20% (CSL Behring, Marburg, Germany). While 10,000 I.U. of heparin were added to the solution for MiECC, only 2500 I.U. were necessary for cCPB.

### 2.3. Sample Processing

Blood was collected at six time points: immediately after induction of anesthesia (T1); 15 (T2) and 60 (T3) minutes after the commencement of CPB, respectively; and 15 (T4) and 120 (T5) minutes after the end of CPB. Last, blood was drawn with the routine laboratory controls at the early morning of the first postoperative day (T6). Blood was collected in ethylenediaminetetraacetic acid (EDTA) tubes (approximately 20 mL) by using the arterial line. Plasma samples were stored at −80 °C and were only thawed once for analyses of the primary study. Clinical and laboratory data were obtained from the patient data management system (IMESO GmbH, Giessen, Germany).

### 2.4. Quantitative Polymerase Chain Reaction

MtDNA encoding for NADH dehydrogenase 1 (ND1) displays a well-described mitochondrial target gene and has already been used as a surrogate marker for the description of mitochondrial damage, and was, therefore, chosen as the target for this secondary analysis [20,21,22,23].

Quantification of ND1 mtDNA was performed by quantitative PCR (qPCR), as previously described [20,23,24]. First, EDTA tubes were centrifuged to separate the plasma (200 g, for 10 min at room temperature), followed by a 1:1 dilutional step (each 100 µL plasma and phosphate-buffered saline (PBS)) and centrifugation at 5000 g (10 min at 4 °C). The supernatant was frozen at −20 °C. After thawing, a commercial purification kit was used for purification of mtDNA according to the manufacturer’s instructions (QIAquick PCR Purification Kit, Qiagen, Venlo, Netherlands). Before qPCR analysis, the samples were diluted 1:20 with nuclease-free, deionized-distilled H_2_O. A StepOnePlus cycler (Thermo Fisher, Waltham, MA, USA) was used for all samples. The ND1 mtDNA primers used were as follows: “ND1 mtDNA FW: 5′-CCA CCT CTA GCC TAG CCG TTT A-3′” and “ND1 mtDNA RW: 5′-GGG TCA TGA TGG CAG GAG TAA T-3′” (synthesized by Eurofins, Luxembourg, Luxembourg).

Mean values of triplicate measurements were calculated for further analysis. As previously described, standard curves were used to convert the results to copies/µL [25]. Standard curves were generated with a plasmid containing human ND1 mtDNA (OriGene Technologies, Rockville, MD, USA) and by the calculation of the number of plasmid copies with a spectrophotometer (NanoDrop 2000, Thermo Fisher Scientific, Waltham, MA, USA). Serial dilutions of the corresponding copy number of plasmids (30–300,000 copies per PCR reaction) were used.

### 2.5. Statistical Analysis

Median and interquartile range (IQR) were used for the description of non-parametric data. To reflect the high variance of data, further analyses were carried out based on the logarithmized data set. General differences in mtDNA quantities were calculated by ANOVA, followed by Tukey’s HSD test for the detection of statistically relevant changes between the study groups with respect to the blood sampling time points. A *p*-value of *p* < 0.05 was considered as statistically significant.

Correlations between mtDNA levels and various parameters were analyzed by linear regression analysis. Outcome parameters were assessed over all time points and at the single time points. Cut-off values for the amount of mtDNA were determined by analysis of the area under the receiver operating curve (AUROC). The resulting cut-off values were subsequently assessed with Fisher’s exact test to define the odds ratios, sensitivity, and specificity. Experimental data, laboratory routine data, and clinical data were stored in an external database (Microsoft Excel, Redmond, WA, USA). Data were analyzed using statistical software R version 4.0.4 (www.r-project.org, accessed on 15 February 2021).

## 3. Results

A total of 45 patients were included in the analysis, of which 23 underwent cCPB and 22 MiECC, respectively. General characteristics are shown in Table 1. Regarding their general characteristics, no significant differences could be detected between the study groups.

### 3.1. Quantification of Free-Circulating ND1 mtDNA Plasma Levels

#### 3.1.1. Time Course

**Figure 1:**
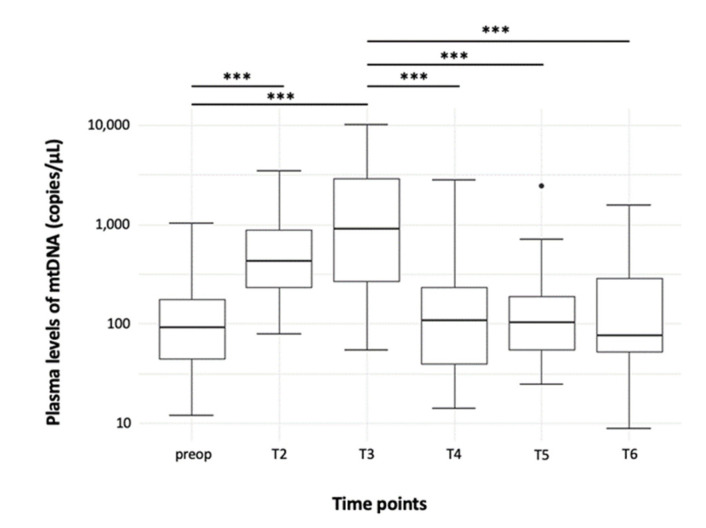
Time course of the amount of mtDNA in peripheral plasma probes (independently of the study group). Results are shown in boxplot diagrams. The black dot at T5 represents an outlier. Asterisks display the degree of statistical significance: ***: *p* < 0.001. Abbreviations: mtDNA: Mitochondrial DNA.

The amount of free-circulating mtDNA rose quickly after the commencement of CPB and significantly peaked 60 min later (Figure 1; Table 2, T1 vs. T2: *p* < 0.001; T1 vs. T3: *p* < 0.001; T2 vs. T3: *p* = 0.006). After completion of CPB, plasma levels of mtDNA decreased rapidly and reached the level of preoperative values again (Figure 1; Table 2, T3 vs. T4: *p* < 0.001; T3 vs. T5: *p* < 0.001; T3 vs. T6: *p* < 0.001; T1 vs. T4, T5, and T6: *p* = 0.88).

#### 3.1.2. Comparison of mtDNA Levels between MiECC and cCPB

**Figure 2:**
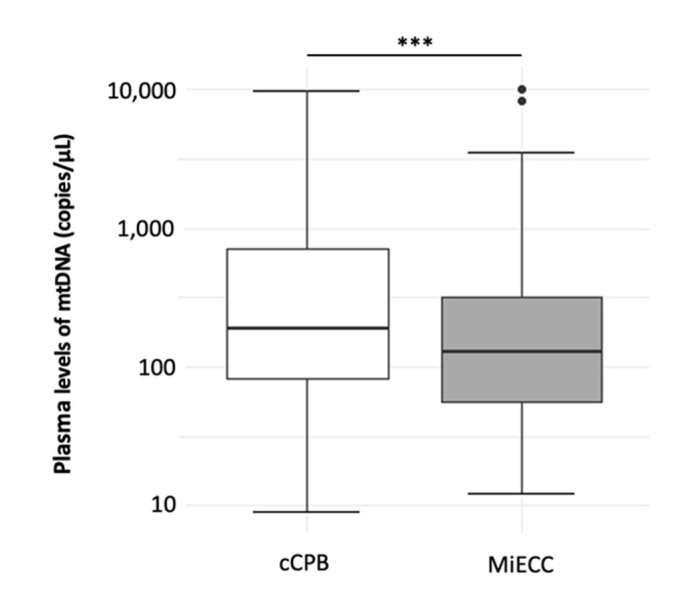
Comparison of both study groups (all time points summarized). Results are shown in boxplot diagrams. Black dots represent outliers. Asterisks display the degree of statistical significance: ***: *p* < 0.001. Abbreviations: cCPB: conventional Cardiopulmonary Bypass; MiECC: Minimized Extracorporeal Circulation.

Overall, the plasma amount of mtDNA was significantly lower in patients undergoing cardiac surgery with MiECC compared to cCPB (Figure 2, MiECC: 161.8 (65.5–501.9); cCPB 190.8 (82–705.7); *p* < 0.001).

**Figure 3:**
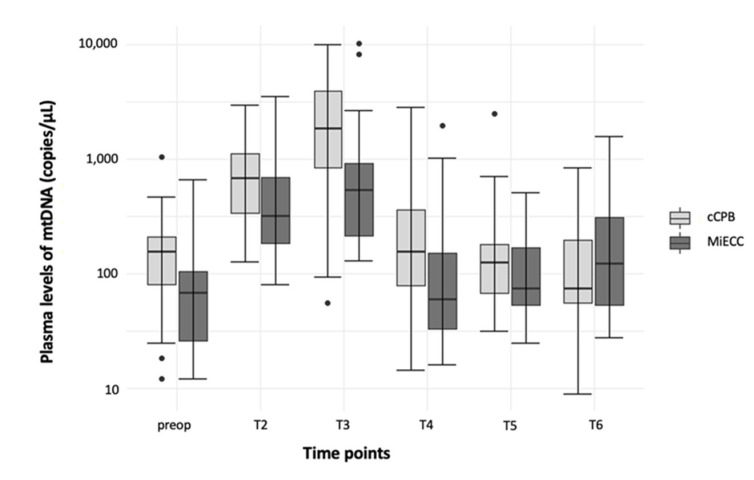
Time course of mtDNA plasma levels depending on the study groups. Results are shown in boxplot diagrams. Black dots represent outliers. Abbreviations: cCPB: conventional Cardiopulmonary Bypass; MiECC: Minimized Extracorporeal Circulation; mtDNA: mitochondrial DNA.

Plasma levels of mtDNA showed comparable courses in both study groups and peaked during CPB (Figure 3; Table 2, MiECC: T1 vs. T2: *p* < 0.001; T1 vs. T3: *p* < 0.001; T3 vs. T4: *p* < 0.001; T3 vs. T5: *p* < 0.001; T3 vs. T6: *p* < 0.01; cCPB: T1 vs. T2: *p* < 0.001; T1 vs. T3: *p* < 0.001; T2 vs. T4: *p* < 0.01; T3 vs. T4: *p* < 0.001; T3 vs. T5: *p* < 0.001; T3 vs. T6: *p* < 0.001). Even though the amount of mtDNA tended to be lower in patients with MiECC, the difference with cCPB failed to reach statistical significance in the single time point analysis.

### 3.2. Correlations between Plasma Free-Circulating ND1 mtDNA Levels and Inflammatory and Ischemic Parameters

Overall, the amount of mtDNA did not correlate with the inflammatory parameters C-reactive protein (CRP), procalcitonin, leukocyte count, and fibrinogen (data not shown).

**Figure 4:**
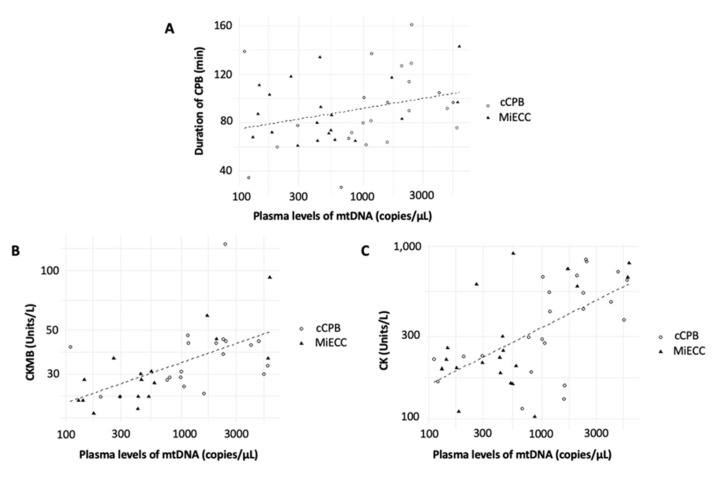
Scatter plot demonstrating the association between the amount of mtDNA and the duration of CPB (**A**), the postoperative blood levels of CKMB (**B**) and CK (**C**), respectively. Abbreviations: CPB: Cardiopulmonary Bypass; cCPB: conventional Cardiopulmonary Bypass; CK: Creatinine Kinase; CKMB: Creatine Kinase Myocardial Band; MiECC: Minimized Extracorporeal Circulation; mtDNA: mitochondrial DNA.

The duration of the CPB was positively correlated with the amount of plasma levels of mtDNA during CPB (T2 and T3), but just very narrowly failed to reach statistical significance (Figure 4A, correlation coefficient = 0.3; adjusted *r*^2^: 0.09; *p* = 0.051).

Further, a significant positive correlation between the amount of CPB-associated mtDNA release (T2 and T3) and the postoperative levels of creatinine kinase (CK) and the creatine kinase myocardial band (CKMB) was found (Figure 4B,C, CKMB: correlation coefficient: 0.47; adjusted *r*^2^: 0.20; *p* < 0.01; CK: correlation coefficient: 0.61; adjusted *r*^2^: 0.3; *p* < 0.001). However, the amount of mtDNA during CPB was not associated with postoperative troponin levels (correlation coefficient: 0.24; adjusted *r*^2^: 0.06; *p* = 0.13).

### 3.3. Correlations between Plasma Free-Circulating ND1 mtDNA Levels and Outcome Parameters

The incidence of adverse outcomes did not differ between the study groups (Table 3). In summary, the cumulated amount of plasma mtDNA over all time points did not reveal an association with any of the outcome parameters (data not shown). However, the ROC analysis of the strongly elevated values after the beginning of CPB (T2) offered a possible cutoff at 644 copies/mL (close to the mean amount of mtDNA of 637.2 copies/mL) with an AUC of 0.63. With the dichotomized variable of mtDNA being higher or lower than the rounded 650 copies/mL, being the sufficient prediction of POAF with an AUROC of 0.71 (0.53–071), a specificity of 67% and sensitivity of 75% was reached, respectively. By taking this into account, further analysis showed that patients whose mtDNA levels peaked above 650 copies/µL at time point 2 had a 5-fold higher risk for the development of POAF, whereas this threshold did not show any predictability for complications other than POAF (Table 3).

## 4. Discussion

The release of mtDNA has been connected to several disease entities such as sepsis, surgical trauma, and, in particular, to cardiac surgery [10,13,26]. Nevertheless, its role in cardiac surgery has been investigated by only a limited number of studies, of which none included patients undergoing CPB with MiECC [12,13,17,18,27].

Here, the time course of mtDNA release began with a strong increase directly after the commencement of CPB, followed by a significant decrease after the end of CBP, independent of the type of CPB. While the absolute plasma levels of mtDNA during cCPB were comparable with other studies investigating CABG patients, the kinetics differed from some studies [17,18]. Nevertheless, they were also aggregable with another investigation [28]. The studies by Qin et al., also showed an early mtDNA release, which, however, did not peak until 12 h after surgery [17,18]. At that time point, the plasma levels of mtDNA almost normalized in our study population. Work conducted by Sandler et al., showed an even later increase on the first postoperative day. However, since they used mtDNA encoding for human cytochrome B, their study results are hardly comparable to ours [13]. On the other hand, analogous to our findings, Baysa et al., reported an early mtDNA peak already occurring 20 min after the start of CPB, indicating that the CPB is mainly responsible for the mtDNA increase [28]. From a physiological point of view, a rapid decrease in mtDNA after the end of CPB is explainable given its fast degradation [29,30,31]. However, it has to be emphasized that the degradation of mtDNA during stress responses is not well understood, and the underlying data is dervied mainly from animal experiments [29,30]. In particular, the potential influence of the CPB on mtDNA integrity has not yet been investigated. Furthermore, these contrasting results could also be explained by other reasons. First, it must be noticed that a direct comparison of mtDNA plasma levels remains challenging due to a lack of standardization. Most studies use methods for relative quantification of mtDNA [12,13] while our study performed an absolute measurement by using plasmid-based qPCR. Second, it is still under discussion whether the surgical incision, specifically the sternotomy, might also contribute to mtDNA release. For example, Naase et al., were able to detect a 19-fold increase in circulating plasma mtDNA after sternotomy in a rodent model [12]. Different surgical techniques might therefore have contributed to a stronger mtDNA release in our study cohort. Third, no details on the used CPB techniques have been reported in previous studies, which might have influenced the amount of released mtDNA. For example, the use of a hemofilter could influence the quantified amount of mtDNA during the CPB. Last, even though both studies by Qin et al., also investigated mtDNA encoding for ND1, they also included only a limited number of patients (38 and 68 patients, respectively) [17,18]. This displays a problem when it comes to biomarkers with a high degree of data variance, as we have observed in our measurements. As a consequence, a high number of patients are necessary to define normal values and kinetics [32,33]. Further, it might limit the mtDNA’s predictive power (e.g., as a biomarker for the prediction of POAF) because a high variance in data points can lead to a reduced specificity, which is well known from sepsis research, for example [32,33].

This study was able to confirm its main hypothesis by showing that the amount of free-circulating mtDNA was higher in patients undergoing cCBP compared to MiECC. To the knowledge of the authors, it is the first to describe this effect of MiECC. However, this finding was only verifiable in the pooled data of all time points. Although the single time point analyses showed a trend toward reduced mtDNA release, the level of statistical significance was not reached. This might be caused for two reasons. First, since this study was designed as an explorative study, a secondary analysis of a pre-existing study was used for data analysis. To exclude potential bias from the study, the investigators chose to eliminate the interventional study group of the primary study, which has been hemodynamically optimized with a goal-directed treatment protocol. As a consequence, the reduced study population might have caused the lacking statistical significance. Second, as mentioned above, the amount of measured mtDNA varied highly with a high proportion of overlapping interquartile ranges. This is particularly remarkable at the preoperative time point because the median of MiECC is lower compared to cCBP, whereas its interquartile range exceeds the corresponding values of cCBP. Further, it must be recognized that patients who suffered from a myocardial infarction immediately or in the last 90 days before surgery were more prevalent in the cCPB group. Even though, the EuroSCORE and other pre-existing diseases did not differ significantly between the study groups, the acute inflammatory state associated with myocardial infarctions might explain the preoperative elevation of mtDNA levels in the cCPB group (which, however, did not reach statistical significance). Nevertheless, these results are supported by studies investigating other inflammatory cytokines and DAMPs. The majority of relevant studies showed an MiECC-induced decrease in inflammatory reactions, which was quantified with various inflammatory biomarkers [6]. For example, Mazzei et al., and Formica et al., showed that the postoperative amount of IL-6, particularly inflammatory patterns of endothelial activation, did not differ between MiECC and off-pump cardiac surgery [7,8]. On the other hand, one randomized controlled trial was not able to identify an MiECC-associated reduction in IL-6, while another revealed an MiECC-induced increase in IL-6 after the first 24 postoperative hours [34,35]. In sum, it is still under discussion whether the proposed anti-inflammatory effects, which are also supported by this study, are relevant to the patients’ outcome.

For this reason, this study aimed, with its secondary hypothesis, to identify potential relations between the amount of mtDNA and commonly assessed outcome parameters in cardiac surgery. This study showed neither a connection between the mtDNA plasma concentration and AKI nor postoperative infections, particularly the readmission rate to the intensive care unit. While the analysis of all time points also did not show a relationship between the levels of mtDNA and POAF, the single time point analysis revealed a significant association with POAF at the early stage of CPB that was independent of the study group. In detail, the risk for POAF was a 5-fold increase when the amount of mtDNA was raised above 650 copies/µL. At this time point, the concentration of mtDNA offered moderate predictive power for the detection of POAF, which is in line with other studies [13,36]. Nevertheless, it must be interpreted with caution because the odd’s ratio’s confidence interval was high because of the above-mentioned high variance of the data and the limited number of included patients. Further, it must be emphasized that this study was not planned for outcome analysis and might therefore be underpowered. However, this result is supported by the study of Sandler et al., who showed with an even smaller number of included patients a connection between mtDNA release and POAF (*n* = 16, [13]). The only available observational study with a higher number of patients (*n* = 485) investigated off-pump cardiac surgery and is therefore hardly comparable [36]. Because of the fact that no CPB was applied during off-pump surgery, the absolute amount of the mtDNA was much lower than in our study, and moreover, another primer was used for qPCR. Nevertheless, even though this study showed only a minor increase in mtDNA, it still offered comparable predictive value for the identification of POAF, supporting our study results (AUROC 0.81; specificity 80.2%; sensitivity 70.3%). However, a recent study that investigated the mtDNA concentration in the blood and pericardial fluid was not able to identify plasma mtDNA as a risk factor for POAF [37]. Interestingly, in this study, patients with POAF presented a significantly higher amount of mtDNA in the pericardial fluid, indicating that it might contribute to the development of cardiac inflammation and consecutive POAF. In contrast, recent studies indicated that mtDNA originates from stressed atrial cardiomyocytes [38,39]. However, this question must be further investigated because it seems that the mtDNA release is also associated with myocardial damage, as indicated by its positive correlation with the postoperative concentration of CK and CKMB in our study. Since our results also hint toward a CPB-time-dependent increase in mtDNA plasma levels, a combination of surgery- and CPB-associated inflammatory reactions with consecutive mtDNA releases seems possible. It will be of high interest to clarify the source of mtDNA in order to evaluate its use as a prognostic biomarker of POAF. Further, the different types of cardiac surgery including MiECC, cCPB, and off-pump techniques must be considered.

This study features some limitations. First, due to its study design as a secondary analysis, no sample size calculation was feasible, which might have led to underpowered study results, particularly regarding the difference between cCPB and MiECC at the single time points. Second, this study is an explorative study, indicating that no conclusions can be drawn regarding the underlying mechanisms of the mtDNA release. Third, even though the surgical and anesthetic management are highly standardized, it cannot be ruled out that individual deviations in this management might have influenced the study results. Fourth, the study groups differed regarding the number of patients suffering from a myocardial infarction recently or in the last 90 days before surgery, with a higher prevalence in the cCPB study group. Since myocardial infarction leads to a high degree of cell damage and inflammation, it might have influenced the preoperative levels of mtDNA in the cCPB study group. Due the design of the study, this bias could not be excluded, but it should be ruled out in future studies. Last, it has to be considered that mtDNA can also be released by arteriosclerosis itself [40]. It must be questioned to what degree it might influence mtDNA release during CPB. The assumption of an influence of arteriosclerosis might explain the high variance inof the measured mtDNA results, especially at the preoperative time point. It might also impact the predictive value of mtDNA because the arteriosclerosis-induced mtDNA release could lead to a high sensitivity but low specificity.

## 5. Conclusions

First, this study was able to show that the overall amount of mtDNA plasma levels was lower in patients undergoing cardiac surgery with MiECC compared to those with cCPB. Even though this trend was also visible in the single time point analysis, this study failed to reach statistical significance due to a high variance in its data and a limited number of included patients. Second, this study identified a positive correlation of the amount of mtDNA with the duration of CPB and postoperative myocardial injury in terms of the increase in CK and CKMB. Last, the early increase in mtDNA after the commencement of the CPB was associated with POAF. At this time point, the amount of mtDNA showed moderate predictive power for the detection of POAF.

## Figures and Tables

**Table 1:** General characteristics.

Parameters	MiECC	cCPB	All Patients
Age (Years)	62 (56–70)	67 (59–73)	65 (57–73)
Male Sex	21/22 (95.5)	17/23 (73.9)	38/45 (84.4)
BMI (kg/m^2^)	29 (28–31)	28 (24–32)	29 (25–32)
EuroSCORE	0.88 (0.72–1.25)	0.99 (0.74–1.2)	0.96 (0.73–1.24)
Pre-existing Diseases			
Angina Pectoris	15/22 (68.1)	15/23 (65.2)	30/45 (66.7)
Arterial Hypertension	21/22 (95.5)	19/23 (82.2)	40/45 (88.9)
Acute Myocardial Infarction	3/22 (13.6)	5/23 (21.7)	8/45 (17.8)
Myocardial Infarction Within the Last 90 Days Prior to Surgery	3/22 (13.6)	8/23 (34.8)	11/45 (24.4)
Concurrent Valvular Disease	6/22 (27.2)	1/23 (4.3)	7/45 (15.6)
Stroke	2/22 (9.1)	3/23 (13.0)	5/45 (11.1)
Chronic Kidney Disease	1/22 (4.5)	3/23 (13.0)	4/45 (8.9)
Diabetes	9/22 (40.9)	6/23 (26.1)	15/45 (33.3)
Chronic Obstructive Pulmonary Disease	2/20 (10.0)	2/23 (8.7)	4/45 (8.9)
Smoker	12/22 (54.5)	14/23 (60.9)	26/45 (57.8)
Alcohol Abuse	6/22 (27.3)	8/23 (34.8)	14/45 (31.3)
Process Times			
Duration of Anesthesia (min)	275 (251–322.0)	279.8 (248.5–320.3)	277.4 (249.5–321.6)
Duration of CPB (min)	83 (68–103)	90 (70–110)	85 (68–107)
Duration of Invasive Ventilation (h)	13.8 (10–16.7)	11.2 (9.4–15.3)	12.5 (9.6–16.5)

Description of the study cohorts. The figures are presented as medians and IQRs (Q25–Q75) or as the number of cases, both the total number and percentage [*n*/*n* total (%)] of the study group. The column “All patients” demonstrates the characteristics of all included study patients. Abbreviations: BMI: Body mass index; CPB: Cardiopulmonary bypass. Abbreviations: BMI = Body Mass Index; CPB = Cardiopulmonary Bypass; IQR = Interquartile Range; MiECC = Minimized Extracorporeal Circulation.

**Table 2:** Amount of plasma levels of mtDNA.

	Plasma Levels of mtDNA (copies/µL)		
Time Points	MiECC	cCPB	All patients
Preoperative (T1)	68.2 (26.5–104.9)	152.5 (80.9–207.6)	91.1 (44.8–175.1)
15 Minutes after Start of CPB (T2)	316.4 (184.2–683.8)	673.0 (334.9–1111.2)	431.7 (235.5–882.4)
60 Minutes after Start of CPB (T3)	536.5 (215.7–919.6)	1818.0 (844.2–3932.2)	904.8 (266.2–2909.3)
15 Minutes after End of CPB (T4)	59.2 (33.2–151.7)	155.0 (79.0–361.8)	108.3 (40.0–232.1)
120 Minutes after End of CPB (T5)	74.6 (52.9–173.0)	124.0 (66.9–182.4)	104.1 (55.6–191.9)
First Postoperative Day (T6)	122.6 (52.6–305.9)	72.9 (56.1–196.6)	75.9 (52.6–287.4)

Results of the quantification of plasma levels of mtDNA. Data are shown as medians and IQRs (Q25–Q75). The column “All patients” demonstrates the plasma mtDNA levels of all included study patients. Abbreviations: CPB: Cardiopulmonary Bypass; mtDNA: Mitochondrial DNA.

**Table 3:** Association between outcome parameters and mtDNA plasma concentrations.

Outcome Parameter	Incidence of Outcome Parameter per Study Group	*mtDNA* > 650 Copies/µL		
		2 × 2 Contingency Table	Odd’s Ratio [CI]	*p*-Value
Acute Kidney Injury	MiECC 3/22 cCPB 1/23	yes 3/4 (75%) no 15/40 (38%)	4.82 (0.35–272.3)	0.29
ICU Readmission	MiECC 2/22 cCPB 1/23	yes 0/3 (0%) no 18/41 (44%)	0 (0–3.46)	0.26
POAF	MiECC 3/22 cCPB 5/23	yes 6/8 (75%) no 12/36 (33%)	5.74 (0.86–66.6)	0.048
Postoperative Infections	MiECC 2/22 cCPB 3/23	yes 2/5 (40%) no 16/39 (41%)	0.96 (0.07–9.41)	1

Outcome parameter analysis at time point 2. Based on the ROC analysis, the cut-off value of 650 copies/µL has been chosen. Incidence is given in the number and percentage of patients with mtDNA higher than the cut-off versus the number of patients per group (outcome yes/no). Data are shown as odds ratios and confidence intervals (CI). Blood collection was missing in one patient at this time point, so therefore, the total number of patients for the examination of mtDNA thresholds is 44. Abbreviations: POAF = postoperative atrial fibrillation.

## Data Availability

The data presented in this study are available on request from the corresponding author.
